# Risk prognosis correlation of insulin-like growth factor binding protein-3 and insulin-like growth factor-1 in uterine fibroids

**DOI:** 10.5937/jomb0-60550

**Published:** 2026-04-15

**Authors:** Tingting Lin, Keke Qian, Yanping Chen, Mengshu Li, Zhang Zhang

**Affiliations:** 1 The People's Hospital of Pingyang, Department of Gynaecology, Wenzhou City, China

**Keywords:** risk and prognosis, uterine fibroids, correlation analysis, immune factor, rizik i prognoza, miom materice, analiza korelacije, imunološki faktori

## Abstract

**Background:**

To explore the relationships between the levels of serum insulin-like growth factor binding protein-3 (IGFBP-3) and insulin-like growth factor 1 (IGF-1) and immune factors, as well as the prognosis of patients with uterine fibroids.

**Methods:**

The study group consisted of 186 patients with uterine fibroids who had laparoscopic myomectomy between June 2023 and June 2024, whereas the control group consisted of 208 healthy women who had physical tests over the same time period. The patients in the study group were divided into a good prognosis group (84 patients) and a poor prognosis group (92 patients). The levels of GFBP-3, IGF-1 and immune factors in the study group and the control group, and their relationships with prognosis were analysed.

**Results:**

Ten patients left the study group, and eight left the control group. IGFBP-3 and IGF-1 in the study group were (3 03.18± 42.39) mg/L and (1377.11 ± 84.78) mg/L, which both outperformed those in the control contingent [(231.2 5 ± 3 4 .1 8) mg/L and (4 3 8 .0 9 ± 5 2 .1 5) mg/L] (t= 12.87, 19.63) P&lt; 0.001). Whereas the levels of CD3+, CD 4+, and CD 4+/CD 8+ were lower than those in the control group (t= 7.92, 8.41, 5.21, P&lt; 0.001). Both values were higher [(284.63± 36.19) mg/L and (4 34.91± 53.28) mg/L]. P&lt;0.001 (t= 5.96, 64.19). While the numbers of CD 3+, CD 4+, and CD 4+/CD 8+ cells were lower than those in the good prognosis group (t= 5.31, 7.03, 3.15). There was a positive correlation between CD8+ status and IGFBP-3, as well as history of uterine fibroids, number of fibroids, history of miscarriage, and duration of breastfeeding (r= 0.593, 0.452, 0.446, 0.419, 0.422, respectively). IGF-1 was negatively correlated with CD 3+, CD 4+, and CD 4+/CD 8+ (r=-0.720, -0.751, and -0.712, respectively). IGF-1 was positively correlated with CD8+ status, history of uterine fibroids, number of fibroids, history of miscarriage, and duration of breastfeeding (r= 0.631, 0.503, 0.444, 0.501, 0.451). The number of fibroids, a history of miscarriage, and the length of breastfeeding were risk variables that impacted the prognosis of patients with uterine fibroids.

**Conclusions:**

The levels of IGFBP-3, IGF-1 and CD-8+ in patients with uterine fibroids are relatively high. Moreover, IGFBP-3 and IGF-1 are related to immune factors and patient prognosis.

## Abstract (sr)

**Uvod:**

Cilj je bio da se ispita povezanost nivoa serumskog proteina koji vezuje insulinu sličan faktor rasta 3 (IGFBP-3) i insulinu sličnog faktora rasta 1 (IGF-1) sa imunoloskim faktorima, kao i sa prognozom kod pacijentkinja sa miomom materice.

**Metode:**

U istraživanje je uključeno 186 pacijentkinja sa miomom materice koje su podvrgnute laparoskopskoj miomektomiji u periodu od juna 2023. do juna 2024. godine, dok je kontrolnu grupu činilo 208 zdravih žena koje su u istom periodu imale sistematske preglede. Pacijentkinje iz posmatrane grupe su podeljene na grupu sa dobrom prognozom (84 pacijentkinje) i grupu sa losom prognozom (92 pacijentkinje). Analizirani su nivoi IGFBP-3, IGF-1 i imunoloskih faktora u posmatranoj i kontrolnoj grupi, kao i njihova povezanost sa prognozom bolesti.

**Rezultati:**

Iz posmatrane grupe je isključeno 10 pacijentkinja, a iz kontrolne 8. Srednje vrednosti IGFBP-3 i IGF-1 u posmatranoj grupi iznosile su (303,18 ± 42,39) mg/L i (1377,11± 84,78) mg/L, sto je značajno vise u odnosu na kontrolnu grupu [(231,25 ± 34,18 ) mg/L i (438,09± 52,15) mg/L] (t= 12,87; 19,63; P&lt; 0,001). Nivoi CD 3+, CD4+ i CD 4+/CD 8+ celija bili su niži nego u kontrolnoj grupi (t= 7,92; 8,41; 5,21; P&lt; 0,001). Obe vrednosti su bile vise [(284,63± 36,19) mg/L i (434,91± 53,28) mg/L; P&lt; 0,001; t= 5,96; 64,19)], dok su brojevi CD 3+, CD4 + i CD 4+/CD 8+ celija bili niži nego u grupi sa dobrom prognozom (t= 5,31; 7,03; 3,15). Utvrđena je pozitivna korelacija između CD8+ statusa i IGFBP-3, kao i sa anamnezom mioma materice, brojem mioma, anamnezom spontanih pobačaja i dužinom dojenja (r= 0,593; 0,452; 0,446; 0,419; 0,422). IGF-1 je bio negativno povezan sa CD 3+, CD4+ i CD4+/CD8+ celijama (r =-0,720; -0,751; -0,712), dok je pokazao pozitivnu korelaciju sa CD8+ statusom, istorijom mioma materice, brojem mioma, pobačajem u anamnezi i dužinom dojenja (r= 0,631; 0,503; 0,444; 0,501; 0,451). Broj mioma, pobačaj u anamnezi i trajanje dojenja identifikovani su kao faktori rizika koji utiču na prognozu kod pacijentkinja sa miomom materice.

**Zaključak:**

Nivoi IGFBP-3, IGF-1 i CD8+ celija povišeni su kod pacijentkinja sa miomom materice. Pored toga, IGFBP-3 i IGF-1 su u značajnoj korelaciji sa imunološkim faktorima i prognozom bolesti.

## Introduction

Uterine fibroids (UFs), the most common type of benign reproductive system tumour among women of childbearing age, have a high incidence rate (up to 20-30%) and significantly affect patients' reproductive health, quality of life and medical burden [1][2][3]. Although its pathogenesis has not been fully elucidated, disorders of the estrogen, progesterone and growth factor network are recognised as the core driving factors [4][5]. As the leading IGF carrier in circulation, IGFBP-3 is not limited to controlling IGF-1's bioavailability and prolonging its half-life but also exerts complex biological effects through IGF-dependent and nondependent pathways (such as directly acting on cell receptors or nuclear transcription factors), including potential proapoptotic and antiproliferative effects [6][7][8]. Therefore, in-depth research on the interrelationship and dynamic changes in the ratio between IGFBP-3 and IGF-1 in the occurrence, development and prognosis of uterine fibroids is significant for clarifying the molecular mechanism of the disease, identifying high-risk populations, assessing the risk of disease progression and exploring potential targeted intervention strategies. However, research in this field faces challenges. On the one hand, the regulation of the IGF system is highly complex.

On the other hand, there are significant individual differences among patients (such as age, hormone status, size/location/number of fibroids), and the results of related studies are often inconsistent [9]. Moreover, large-sample, well-designed prospective studies to verify its reliability as a prognostic marker and its clinical application value are lacking [10]. Elevated levels of IGF-1 in circulation or local tissues and abnormal expression of IGFBP-3 are associated with uterine fibroids; their value as independent or combined risk prediction and prognosis evaluation indicators has not been clearly established. In particular, there is a lack of prospective analyses on the dynamic balance and the clinical outcomes of patients (such as the growth rate of fibroids, recurrence risk, and severity of symptoms) [11]. This study aims to systematically analyse the changes in the levels, fibroid burden, and key prognostic indicators (such as postoperative recurrence and symptom relief), with the expectation of clarifying their potential value in the risk assessment and prognosis prediction of uterine fibroids [12][13][14]. This study provides a new molecular basis for individualised disease management and precision medicine.

Uterine fibroids can lead to immune dysfunction within the body, preventing it from regulating the trauma suffered by the body and affecting its recovery. Moreover, as the severity of a patient's condition increases, immune disorders within the body intensify, impacting the clinical treatment outcome. IGFBP-3 and IGF-1 in uterine fibroids were examined, providing a reference for the prevention and treatment of the poor prognosis of patients with uterine fibroids.

## Materials and methods

### Clinical data collection

A case-control investigation was carried out. From June 2023 to June 2024, 186 individuals with uterine fibroids who underwent laparoscopic myomectomy at our institution were selected for the study. It was 32.76±4.87 years old on average, the number of deliveries was 2.06±0.37, and the number of pregnancies was 2.13±0.46. The duration of the breastfeeding period was 6.19±1.03 months. The control group consisted of an additional 208 healthy women who were examined during the same time period. The average age was 32.83±4.89 years, the number of deliveries was 1.98±0.31, the number of pregnancies was 2.19±0.51, and the lactation time was 6.26±1.06 months.

### Inclusion criteria

The patient met the diagnostic criteria for uterine fibroids as stipulated in the International Journal of Obstetrics and Gynaecology. B-ultrasound examination confirmed the presence of uterine fibroids. All patients underwent surgical treatment; the patients presented short strip-like, long strip-like and ringshaped blood flow signals.

### Exclusion criteria

Malignant tumours such as breast cancer and cervical cancer; abnormal heart, liver and kidney functions; coagulation dysfunction; concurrent infectious diseases; mental illness; pregnancy or lactation; ovarian dysfunction; severe anaemia; and incomplete medical record data.

Patients whose conditions changed during the research process; those who experienced serious adverse events and were no longer suitable for treatment, and patients or their close relatives who requested terminating the study and withdrew voluntarily.

### Surgical methods of the research group

Laparoscopic myomectomy was performed. General anaesthesia was administered. A bladder lithotomy position was taken, and a uterine lift device was placed. Artificial pneumoperitoneum was established via the three-port puncture method. The patient's abdominal pressure was 12 mmHg (1 mmHg = 0.133 kPa). A trocar with a 10 mm endoscope was placed. Laparoscopy was used to explore the location of the uterine fibroids, and surgical treatment was performed at different locations. Broad ligament fibroids: Explore the tumour nucleus and the ipsilateral ureter, open the anterior lobe of the broad ligament, and obtuse separate the fibroid tissue. Cervical fibroids: The position relationships among the bladder, rectum and fibroids were observed. The bladder or rectum was pushed down, and the peritoneum was folded back to expose the fibroids. Posterior pituitary hormone was injected at the junction of the fibroids and the serosal layer, and uterine oxytocin was simultaneously administered intravenously. A monopolar electrohook was used to cut the fibroid capsule, exposing the fibroids. The fibroids were pulled under toothed forceps, electrocoagulated and disconnected, and the surface tissue of the fibroids was passively separated. The positional relationships among the bladder, the lower segment of the uterus and the fibroids were observed. After injecting posterior pituitary hormone, the surface of the fibroids was cut, the tumor nuclei were exposed, and the fibroids were removed. After the operation, routine irrigation was performed, and a drainage tube was indwelled. The operation was completed.

### Index detection

### Detection of IGFBP-3 and IGF-1

Centrifugation speed 3000 r/min, centrifugation time 10 min, and the supernatant was collected and maintained at -80°C. The enzyme-linked immunosorbent assay (ELISA) [IGFBP-3: ELISA-No.EHIGFBP3, Thermo Fisher, The United States; IGF-1: ELISA-No.EH250RB, Thermo Fisher, The United States] was used to determine the levels of IGFBP-3 and IGF-1.

To accurately quantify the concentrations of these two biomarkers in the cycle, this study employed the enzyme-linked immunosorbent assay (ELISA) for detection. All the samples to be tested were derived from the peripheral venous blood of the study participants. Considering the standardisation requirements of the detection methods, the stability of biomarkers and their broad applicability, we uniformly chose serum as the measurement matrix. Blood collection strictly follows standardised procedures: serum separation, using vacuum blood collection tubes containing coagulants and inert separation gels (commonly known as »yellow-headed tubes« or »gold-headed tubes«). After blood collection, the sample is left to stand vertically at room temperature for 30 to 60 minutes until the blood is fully coagulated. Then, it is centrifuged under standardised conditions (4°C, 3000 rpm for 10 minutes). The upper layer of separated serum was carefully transferred to a sterile cryotube, avoiding contact with the separation gel or blood cell layer, and immediately stored at -80°C until batch ELISA testing was conducted.

### Detection of immune factors

All the subjects had 8 mL of fasting venous blood drawn. The levels of T lymphocyte differentiation antigen molecule 3+ (CD3+), T lymphocyte differentiation antigen molecule 4+ (CD4+), and T lymphocyte differentiation antigen molecule 8+ (CD8+) were detected via flow cytometry. The BD FACSCanto™ II flow cytometer was used for determination, and the concentrations of insulin-like growth factor-binding protein-3 (IGFBP-3) and insulin-like growth factor-1 (IGF-1) in the samples were measured using the highly sensitive ELISA kit provided by R&D Systems. The absorbance was measured using the Multiskan™ GO microplate reader produced by Thermo Fisher Scientific.

In terms of sample processing, we adopted a combined method of manual and automatic washing. The laboratory personnel manually carry out the initial processing and addition steps of the samples to ensure the accuracy and consistency of the operation. During the plate washing process, we used the ELx50 automatic plate washer produced by BioTek.

### Clinical data collection

The general information of the subjects, including age, body mass index (BMI), history of cesarean section (yes or no), history of hypertension (yes or no), family history of uterine fibroids (yes or no), number of fibroids (single or multiple), type of fibroids (intramural fibroids, submucosal fibroids, subserosal fibroids, sarcoma), history of miscarriage (yes or no), and duration of breastfeeding (< 6 months or ≥ 6 months), was collected.

### Prognostic observation

Telephone calls were used to follow up with the study group's patients for a year, including outpatient visits and other related activities. When a patient passed away during the follow-up period, the followup was stopped. The growth, metastasis and recurrence of fibroids during the follow-up period were statistically analysed.

### Statistical methods

The statistical software program SPSS 19.0 was used for the statistical analysis. The measurement results, which are shown as means±s, were distributed regularly. Group comparisons were performed using two independent samples t-tests. Percentages are used to represent the count data. The χ^2^ test was employed to compare groups, and Spearman correlation analysis was used to examine the relationship between the variables.

## Results

### Levels of IGFBP-3 and IGF-1 in the study and control groups

Eight patients in the control group stopped participating in the trial, 6 patients withdrew from the study group, and 4 patients experienced changes in their conditions. The study group had considerably higher levels of both IGFBP-3 and IGF-1 compared to the control group (all P<0.001) (Table 1).

**Table 1:** Comparison of Ang-2 and IGF-1 levels between two groups.

Group	Cases	IGFBP-3 (ng/mL)	IGF-1 (mg/L)
Control group	200	231.25±34.18	438.09±52.15
Research Group	176	303.18±42.39	1377.11±84.78
t value	_	12.87	19.63
P-value	_	<0.001	<0.001

The levels of insulin-like growth factor binding protein-3 (IGFBP-3) and insulin-like growth factor-1 (IGF-1) in the serum of patients with uterine fibroids (study group) were significantly higher than those in the healthy control group. Specifically, the average serum IGFBP-3 concentration of the patients in the study group (n = 186) was (303.18±42.39) mg/L, while that of the healthy control group (n = 208) was only (231.25±34.18) mg/L. The difference between the two groups was highly statistically significant (t=12.87, P<0.001). Similarly, the average concentration of serum IGF-1 in the study group was as high as (1377.11±84.78) mg/L, which was much higher than that in the control group [(438.09±52.15) mg/L]. The difference was also highly significant (t=19.63, P<0.001).

### Comparison of immune factor levels between the control group and the study group

The levels of CD3+, CD4+, and CD4+/CD8+ cells were lower in the study group than in the control group, except for CD8+ cells, which were higher. All P<0.001 indicated that the differences were statistically significant (Table 2).

**Table 2:** Immune factor levels between groups (x̄ ± s).

Group	Cases	CD3+ (%)	CD4+ (%)	CD8+ (%)	CD4+/CD8+
Control group	200	53.02±6.25	42.36±5.25	21.36±3.25	1.70±0.23
Research Group	176	46.25±5.34	36.44±4.29	29.36±3.69	1.48±0.35
t value	-	7.92	8.41	-15.79	5.21
P-value	-	<0.001	<0.001	<0.001	<0.001

The key immune indicators of patients with uterine fibroids (study group) were significantly different from those of healthy women (control group). The distribution of T lymphocyte subsets in the study group patients showed significant abnormalities: the levels of CD3+ T cells and CD4+ T cells in their peripheral blood, as well as the CD4+/CD8+ ratio, were significantly lower than those in the healthy control group. Specifically, the levels of CD3+ and CD4+ cells and the ratio of CD4+/CD8+ in the study group (n = 186) were significantly lower than those in the control group (n = 208), and the statistical differences were highly significant (t values were 7.92, 8.41, and 5.21, respectively, all P<0.001). This result suggests that patients with uterine fibroids have immune status changes characterised by cellular immune function imbalance, mainly manifested as a reduction in the number of total T cells (CD3+) and helper/inducer T cells (CD4+), as well as an imbalance in the T cell subset ratio (CD4+/CD8+).

### IGFBP-3 and IGF-1 levels in patients with varying prognoses

The bad prognosis group's IGFBP-3 and IGF-1 levels were both noticeably higher than those of the good prognosis group (all P<0.001) (Table 3).

**Table 3:** IGFBP-3 and IGF-1 levels between patients with uterine fibroids (x̄ ± s).

Group	Cases	IGFBP-3 (ng/mL)	IGF-1 (mg/L)
Good prognosis group	84	284.63±36.19	434.91±53.28
Poor prognosis group	92	335.16±42.67	1406.18±83.77
t value	—	5.96	64.19
P-value	—	<0.001	<0.001

The levels of serum IGFBP-3 and IGF-1 in the poor prognosis group were significantly higher than those in the good prognosis group. Specifically, the average concentration of serum IGFBP-3 in the poor prognosis group was (284.63±36.19) mg/L, while the average concentration of IGF-1 was (434.91 ± 53.28) mg/L. Compared with the patients in the good prognosis group, the differences were highly statistically significant (t values were 5.96 and 64.19, respectively, both P<0.001). This discovery clearly suggests that higher levels of IGFBP-3 and IGF-1 in the serum are closely related to the poor prognosis of patients with uterine fibroids.

### Comparison of immune factor levels in patients with different prognoses

While the level of CD8+ cells was higher than that of the good prognosis group, CD3+, CD4+, and CD4+/CD8+ cell levels were lower in the bad prognosis group (P<0.05) (Table 4).

**Table 4:** Comparison of immune factor levels between patients with uterine fibroids (x̄ ± s).

Group	Cases	CD3+ (%)	CD4+(%)	CD8+ (%)	CD4+/CD8+
Good prognosis group	84	6.25±5.413	6.25±4.19	22.25±3.66	1.40±0.21
Poor prognosis group	92	40.36±5.02	30.11±4.02	30.98±4.27	1.27±0.18
t value	-	5.31	7.03	-10.27	3.15
P-value	-	<0.001	<0.001	<0.001	0.002

The number of CD3+ T cells, CD4+ T cells and the ratio of CD4+/CD8+ in the peripheral blood of patients in the poor prognosis group were significantly lower than those in the good prognosis group, and the differences were statistically significant (t values were 5.31, 7.03 and 3.15, respectively). This finding indicates that patients with a poor prognosis have lower levels of total T cells (CD3+) and helper T cells (CD4+), and the imbalance of T cell subsets (reflected by the CD4+/CD8+ ratio) is more severe, suggesting a stronger suppression or disorder of cellular immune function. This difference in immune status is highly correlated with a poor prognosis for patients. In addition, this study also found that the serum IGF-1 level was significantly negatively correlated with CD3+, CD4+ and the ratio of CD4+/CD8+ (r =-0.720, -0.751, -0.712), while the IGF-1 level was significantly increased in the poor prognosis group.

### Correlations between IGFBP-3, IGF-1 and immune factors and prognosis

Correlation analysis revealed that while IGFBP-3 and IGF-1 were positively connected with CD8+ status and uterine fibroids history, they had a negative correlation with CD3+, CD4+, and CD4+/CD8+ status, number of fibroids, history of miscarriage, and duration of breastfeeding (all P<0.05) (Table 5).

**Table 5:** Correlation analysis of IGFBP-3, IGF-1 and the prognosis of immune factors in patients with uterine fibroids.

Indicator	CD3+		CD4+		CD8+		CD4+/CD8+	
	r value	P-value	r value	P-value	r value	P-value	r value	P-value
IGFBP-3	-0.623	0.014	-0.578	0.012	0.593	0.023	-0.662	0.004
IGF-1	-0.720	0.001	-0.751	0.001	0.631	0.010	-0.712	0.001
Indicator	History of uterine fibroids		The number of fibroids		History of miscarriage		Breastfeeding time	
	r value	P-value	r value	P-value	r value	P-value	r value	P-value
IGFBP-3	0.452	0.001	0.446	0.001	0.419	0.001	0.422	0.001
IGF-1	0.503	0.001	0.444	0.001	0.501	0.001	0.451	0.001

Correlation analysis indicated that IGF-1 was systematically negatively correlated with key cellular immune indicators: its level was highly significantly negatively correlated with CD3+ T cells, CD4+ T cells, and the ratio of CD4+/CD8+ (correlation coefficients r were -0.720, -0.751, and -0.712, respectively; all P<0.001). On the contrary, IGF-1 was significantly positively correlated with the CD8+ status (r=0.631). IGFBP-3 was also positively correlated with the CD8+ status (r=0.593). These results collectively indicate that the elevated levels of serum IGF-1 and IGFBP-3 are closely related to the imbalance of cellular immune function in patients, characterised by the relative increase of inhibitory/cytotoxic T cells (CD8+). However, total T cells (CD3+), helper T cells (CD4+), and the immunomodulatory balance (the ratio of CD4+ to CD8+) were inhibited.

### Risk factors for the prognosis of patients with uterine fibroids, multivariate logistic regression

The statistically significant indicators were taken as independent variables (no family history of uterine fibroids =0, with a family history of uterine fibroids = 1; single fibroids =0, multiple fibroids =1; no history of miscarriage =0, history of miscarriage =1; breastfeeding duration ≥ 6 months =0, breastfeeding duration < 6 months =1). The family history of uterine fibroids, the number of fibroids, a history of miscarriage, and breastfeeding time were risk variables that impacted the prognosis of uterine fibroids patients (all P<0.05) (Table 6).

**Table 6:** Multivariate logistic regression analysis of prognostic risk factors in patients with uterine fibroids.

Factor	β value	SE value	Wald x^2^ value	P-value	OR value	95% CI
Family history of uterine fibroids	1.421	0.336	17.89	0.001	4.141	3.469~4.813
The number of fibroids	1.296	0.225	33.18	0.001	3.655	3.205~4.105
History of miscarriage	1.332	0.421	10.01	0.001	3.789	2.947~4.631
Breastfeeding time	1.214	0.332	13.37	0.001	3.367	2.703~4.031

## Discussion

Uterine fibroids are benign tumours, and if not diagnosed in time, they can cause calcification changes and promote continuous growth, leading to tumour deterioration and threatening patient safety [15].

Among the most prevalent is insulin-like growth factor binding protein-3 (IGFBP-3), an intensely studied member of the insulin-like growth factor binding protein (IGFBP) family [16]. It is the main carrier protein of IGF-I and IGF-II, responsible for binding and transporting more than 80% of the IGFs in the circulation [17]. The core function of IGFBP-3 lies in precisely regulating the biological activity and availability (especially IGF-I). By binding high-affinity IGFs, it significantly prolongs the half-life of IGFs in the blood and protects them from degradation. On the other hand, the formation of macromolecular complexes restricts the escape of IGFs from the vascular lumen, effectively reducing the chance of IGFs binding to their receptor (IGF-1 R). This reduces the biological effects of IGFs, such as mitogenic promotion and antiapoptotic effects. In addition, IGFBP-3 can directly act on cells through pathways independent of IGFs (such as binding to its own specific receptors or regulating nuclear transcription factors), exerting biological effects such as inhibiting proliferation and inducing apoptosis. IGF-1 is a single-chain polypeptide factor synthesised and secreted by liver cells that can regulate the cell cycle [18][19][20]. Moreover, IGF-1 can promote the mitotic activity of smooth muscle cells through estrogen, accelerate the cell proliferation rate, and promote the generation of tumour cells. Elevated IGF-1 levels can alter the activity of normal cells, promote cell proliferation and division, and reduce cell death. Patients with uterine fibroids suffering from chronic hyperinsulinemia can have increased serum IGF-1 levels [21]. IGF-1 exerts a special gonadotropin function by stimulating the ovaries to secrete hormones, which is conducive to the occurrence and development of uterine fibroids [22]. Therefore, in clinical practice, the IGF-1 level of patients should be detected promptly to control further progression of the disease in patients with uterine fibroids [23][24].

The occurrence of uterine fibroids can disrupt the immune balance within the body. As the clinical symptoms of uterine fibroids worsen, they can lead to the loss of immune function, cause an abnormal immune system, and result in cancer. When immune function within the body is suppressed, diseased cells can escape the surveillance of immune cells. This promotes the proliferation of tumour cells and leads to the recurrence and deterioration of uterine fibroids, thereby affecting the prognosis of patients [25].

The possible reasons are as follows: a large number of fibroids and their hidden locations can increase the difficulty of surgery and lead to incomplete removal of fibroids during the operation, resulting in the recurrence of fibroids [26][27][28]. After abortion, it can lead to endocrine disorders in the body, increase the secretion of estrogen and increase the incidence of uterine fibroids [29]. Moreover, miscarriage can damage the endometrium. Self-repair of the uterus can increase the level of epidermal growth factor in some parts of the endometrium, leading to uterine fibroids [30]. Breastfeeding can affect hormone levels in the body. Breastfeeding can interrupt the secretion of estrogen in the body and inhibit its promoting effect on uterine fibroids, thereby reducing their incidence [31]. Patients with a poor prognosis can be identified based on prognostic risk factors and serum indicators to control their condition and prevent further progression of the disease.

## Conclusion

The prognosis of patients with uterine fibroids can be affected by the family history of uterine fibroids, the number of fibroids, the history of miscarriage, and the duration of lactation. Moreover, the expressions of IGFBP-3, IGF-1, and CD8+ increase, which are related to the prognosis of patients. Therefore, in clinical practice, patients should be actively treated to control IGFBP-3, IGF-1 and immune factors to enhance the prognosis for patients with uterine fibroids.

## Dodatak

### Authors' contribution

Tingting Lin and Keke Qian contributed equally to this paper.

### Conflict of interest statement

All the authors declare that they have no conflict of interest in this work.
